# A Highly Robust Silicon Ultraviolet Selective Radiation Sensor Using Differential Spectral Response Method †

**DOI:** 10.3390/s19122755

**Published:** 2019-06-19

**Authors:** Yhang Ricardo Sipauba Carvalho da Silva, Rihito Kuroda, Shigetoshi Sugawa

**Affiliations:** Graduate School of Engineering, Tohoku University, 6-6-11-811, Aza-Aoba, Aramaki, Aoba-ku, Sendai, Miyagi 980-8579, Japan; rihito.kuroda.e3@tohoku.ac.jp (R.K.); shigetoshi.sugawa.d4@tohoku.ac.jp (S.S.)

**Keywords:** UV, sensor, ultraviolet, sensing, differential, spectral, response, silicon, photodiode

## Abstract

This paper presents a silicon ultraviolet radiation sensor with over 90% UV internal quantum efficiency (QE) and high selectivity to the UV waveband without using optical filters. The sensor was developed for applications that require UV measurement under strong background visible and near-infrared (NIR) lights, such as solar UV measurement, UV-C monitoring in greenhouses or automated factories, and so on. The developed sensor is composed of monolithically formed silicon photodiodes with different spectral sensitivities: a highly UV responsive photodiode with internal quantum efficiency (QE) of nearly 100% for UV light, and a lowly UV responsive photodiode with UV internal QE lower than 10%. The photodiodes were optimized to match their visible and NIR light responsivity, and the UV signal is extracted from the background radiation by using the differential spectral response method. With this approach, an internal QE of over 90% for UV light was obtained, with a residual internal QE to non-UV light lower than 20% for 400 nm, 5% for 500 nm, 2% for 600 nm and 0.6% to NIR light. The developed sensor showed no responsivity degradation after exposure towards strong UV light. It was confirmed by the simulation results that the residual responsivity is further suppressed by employing an on-chip band-rejection optical layer consisting of several layers of silicon oxide and silicon nitride films.

## 1. Introduction

Ultraviolet (UV) radiation sensing has been extensively researched and applied to several fields including scientific analysis, industrial, medical, safety systems and so on [1,2,3,4]. Some examples of already reported applications are UV spectroscopy [5], UV cure process monitoring [6], solar UV measurement for healthcare applications [7], flame detection [8], semiconductor process control [9], among many others. The target wavelength for those applications are mainly in the UV-A (315 nm to 380 nm), UV-B (280 nm to 315 nm) and UV-C (200 nm to 280 nm) wavebands. Also, far UV (10 nm to 200 nm) and extreme UV (1 nm to 10 nm) wavebands are useful for monitoring of photolithography processes [10]. Due to this broad scope of applications, there is an increasing demand for compact UV sensing devices with high UV light responsivity and high robustness to continuous UV exposure, without responsivity degradation over time. Also, high selectivity of responsivity to UV light is crucially important in the applications that require UV radiation detection under ambient visible and near-infrared (NIR) light, such as solar UV measurement and flame detection. A sensor with a simple and low-cost manufacturing process is also desirable for cost-sensitive applications.

Current UV sensors developed to meet some of those requirements can be classified into four technological categories. The first category is of sensors that employ compound semiconductors with a wide bandgap, such as SiC, AlGaN and GaAsP [11,12,13]; the second category uses a Silicon-On-Insulator (SOI) structure containing a shallow surface detection layer with a thickness of a few tens of nanometers [7]; the third category employs silicon (Si) light detectors with a high concentration surface layer to form drift fields from near the Si surface for achieving high UV responsivity and high robustness to continuous UV light exposure [14,15,16,17]. The fourth category employs sensors with UV and visible responsivity that captures the background visible light in advance and subtracts it from the subsequent detected light [18]. 

In each of those categories, selective detection of UV light is realized by leveraging the high energy of the UV light photons, or the short penetration depth of UV light in silicon. However, those approaches have the drawbacks of making use of special materials such as compound semiconductors and SOI, or utilizing on-chip or off-chip optical filters to cut visible and NIR light responsivity, or needing an environment without non-UV light or at least with a stable visible light background for UV detection. In this work, we aimed to develop a silicon (Si) UV radiation sensor with high UV responsivity and high selectivity to the UV waveband, without employing external bandpass filters and capable of UV detection under variable background visible and NIR light. Silicon photodetectors were used because of the low manufacturing cost and high adaptivity of monolithic integration with external circuits. 

When using Si photodiodes in UV sensing applications, the following two challenges of the conventional Si photodiode technology have to be addressed: first, low UV light responsivity and low robustness to continuous exposure to UV light, with responsivity degradation and dark current increase after exposure [19,20]. Second, low selectivity to the UV waveband, due to the response to visible and NIR wavebands. Approaches to overcome the first issue have already been reported, such as delta-doped charge-coupled device (CCD) image sensors [17], PureB photodiodes [15,16], and silicon photodiodes manufactured with a thin surface layer with high dopant concentration and a steep dopant profile in a flat Si surface [21,22,23]. The mechanism of UV detection in all those approaches is similar: a depletion layer from near the Si surface is formed, drifting the electrons photo-generated within a few atomic layers from the Si surface to achieve high UV responsivity. The high surface concentration has the function of suppressing changes in the electrical drift field caused by fixed charges induced in the dielectric film due to UV exposure, to achieve high robustness to UV light exposure [17,21,22,23]. 

**Figure 1:**
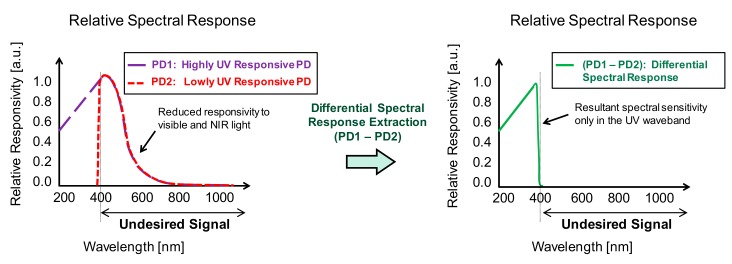
Conceptual diagram of the differential spectral response UV sensor. High responsivity and high selectivity in the UV waveband are obtained by extracting the differential response of the highly UV responsive photodiode PD1 and lowly UV responsive photodiode PD2.

To overcome the second issue, however, a new approach is needed. In this work, we addressed this issue by developing two types of photodiodes: a highly UV responsive photodiode—PD1 and a lowly UV responsive photodiode—PD2, both with matching spectral sensitivity for visible and NIR light. The UV light signal is extracted by using the differential spectral response method [24], as illustrated in Figure 1. The dependence of the light penetration depth on the wavelength was utilized for the design of PD1 and PD2. 

The structure of this paper is as follows. In Section 2, we describe the aspects considered in the photodiode’s design, from simple theoretical calculations of the spectral sensitivity to process and device simulations using the proposed photodiode structures. The process flow and profiles used in sample fabrication are also summarized. In Section 3, the measurement results of responsivity of fabricated PD1 and PD2 are presented, the internal quantum efficiencies (QEs) and the differential spectral response (PD1 – PD2) are calculated and compared with the simulation results. Measurement results of responsivity stability towards UV irradiation stress are also shown. A brief discussion of a band-rejection interference interlayer capable of shaping the developed UV sensor’s spectral sensitivity and suitable for monolithic integration with PD1 and PD2 is also presented. This paper is the extended version of a previous report [24], with detailed design information, newly added simulation and experimental results and with the introduction of the developed on-chip interference interlayers. 

## 2. Sensor Design, Fabrication and Measurement Setup

In this section, the device structure and the internal potentials considered in the design of PD1 and PD2 are described. At first, simple calculations of the internal QE for each photodiode were carried out based on the wavelength dependence of light penetration depth in Si. Those calculations give a guide for the design of internal potentials in PD1 and PD2. After that, we present the process and device simulations performed to calculate the ion implant process conditions, the dopant and carrier concentration profiles for each layer, and the internal QEs of the photodiodes more accurately. Finally, we present the manufactured chips’ conditions. 

### 2.1. Device Structure and Concept

**Figure 2:**
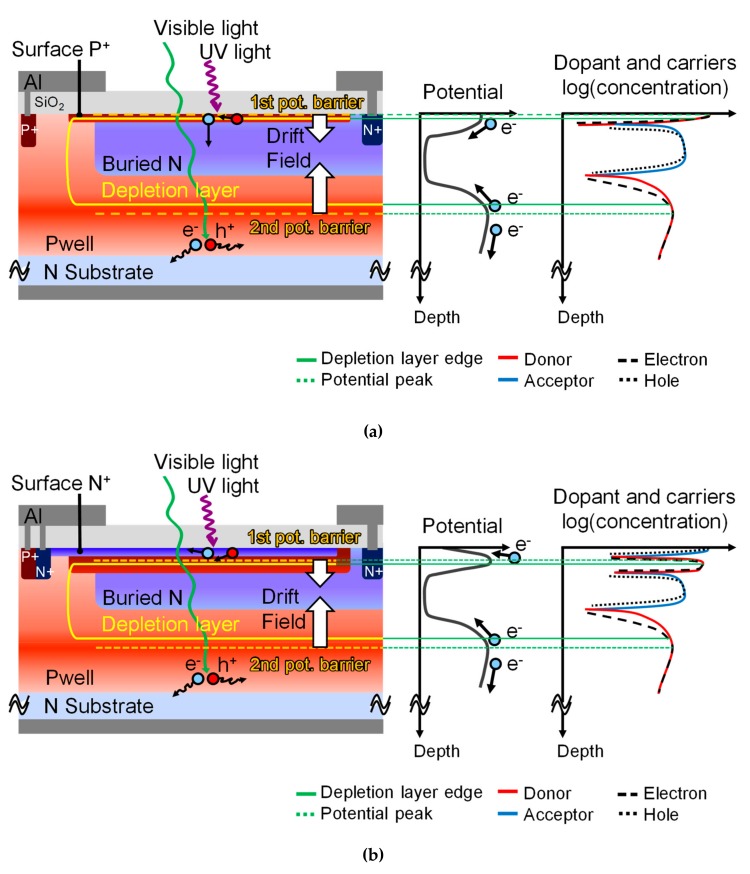
Device structures of the photodiodes (out of scale) (**a**) PD1, with high UV responsivity, and (**b**) PD2, with low UV responsivity. The potential maxima near the surface (first potential barrier) and within the Pwell layer (second potential barrier) are shown. The surface P^+^ and surface N^+^ layers are grounded and the buried N layer is biased at 0.3 V. Also, diagrams for the potential distribution and the concentrations of donors, acceptors, electrons and holes in the depth axis are presented for each type of photodiode.

Figure 2 shows the device structures (out of scale), the internal potential diagrams and the dopant and carriers concentration profiles of PD1 and PD2, for a bias of 0.0 V applied to the surface P^+^ and surface N^+^ layers, and a bias of 0.3 V applied to the buried N layers. PD1 has a similar surface structure to the previously reported photodiode with 190 nm to 1100 nm spectral sensitivity and high robustness to UV light exposure [21,22,23], however a Pwell layer was added to reduce visible and NIR light responsivity. The PD2 structure, when compared with PD1, has an added surface N^+^ layer to drain out and recombine electrons generated near the surface, effectively acting as a layer without responsivity (dead layer). As further explained, this approach is effective to selectively reduce UV light responsivity in the photodiode. PD2 also has a surface P^+^ layer deeper and with higher concentration than PD1, to avoid full depletion of the surface P^+^ layer. This approach is necessary because, if full depletion on the surface P^+^ layer occurs in PD2, leakage current will flow between the surface N^+^ and the buried N layer. Thus, surface P^+^ layer implant conditions of PD1 and PD2 must be individually chosen due to the presence or absence of the surface N^+^ layer in each type. Also, in the low UV responsivity photodiode PD2, both the surface N^+^ and the surface P^+^ layers are set to the same potential level (ground level). The UV signal is obtained by the differential spectral response method instead of directly from the surface N^+^ layer of PD2. Applying a bias voltage in the PD2 surface would pose challenges such as reverse leakage currents, spread of depletion width and high capacitance in PD2. The proposed approach in this paper avoids those drawbacks. 

Regarding the potential diagrams, each type of photodiode has the buried N layer (charges detection layer) comprised of a first and a second potential barrier. The first potential barrier is the potential maxima in the surface P^+^ layer, while the second potential barrier is the potential maxima in the Pwell layer. Those potentials are important because they determine the depth range of generated charges that will be drifted to the buried *N* layer and detected. Due to the very short UV light penetration in silicon, the first potential barrier can be used to adjust UV light responsivity for PD1 and PD2. For PD1 it is set at the surface, and for PD2 it is set at a few tens of nanometers below the surface. Similarly, due to the long penetration depth of visible and mainly NIR wavebands, the second potential barrier can be used to reduce responsivity to those longer wavelengths. The second potential barrier is set at a few hundreds of nanometers below the surface. Reducing the responsivity of PD1 and PD2 to visible and NIR wavebands is useful to reduce undesired signals before the differential signal extraction, for better accuracy. 

### 2.2. Internal QE Estimation Using Light Penetration Depth

**Figure 3:**
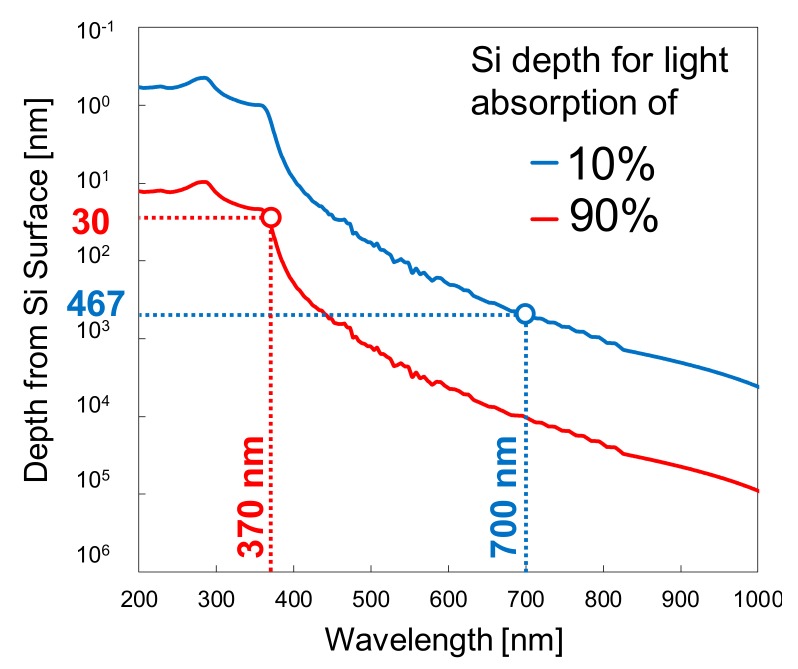
Calculation of the depth from the silicon surface that 10% and 90% of incident light is absorbed, for each wavelength.

When light is irradiated in silicon, light intensity decreases exponentially with the depth, due to light absorption in the photoelectric conversion effect, according to the following equations [25].

(1) $$I=\;I_{0}\times e^{-\left(\alpha \;\times \;d\right)},$$

where

(2) $$\alpha \;=\;\frac{4\;\times \;\pi }{\lambda }\times k,$$

where, *I* is the light intensity at a depth *d* from the surface, *I*_0_ is the light intensity at the surface, *α* is the absorption coefficient, *λ* is the light wavelength and *k* is the extinction coefficient for the wavelength *λ*. Using Equations (1) and (2) and the extinction coefficients of silicon [26], the depths for obtaining 10% and 90% light absorption, as a function of the wavelength, were calculated and are shown in Figure 3 as a blue and a red line, respectively. Figure 3 is useful in the design of PD1 and PD2, for the analysis of the depths necessary for the internal potential barriers (first and second potential barriers of Figure 2). For instance, from the red line in Figure 3, we can conclude that for UV light with wavelengths shorter than 370 nm, 90% of light absorption occurs within 30 nm from the Si surface. Therefore, UV internal QE in PD2 can be reduced by more than 90% by setting the first potential barrier of PD2 at 30 nm or more from the Si surface. Similarly, from the blue line in Figure 3, we conclude that within 467 nm from the Si surface, less than 10% of the incident light with wavelengths longer than 700 nm is absorbed. Therefore, NIR internal QE can be reduced by more than 90% by setting the second potential barrier at 467 nm or less from the Si surface in both PD1 and PD2.

Equations (1) and (2) can also be used to estimate the internal QE and the responsivity of Si photodiodes by assuming that all and only the electrons generated by the light absorbed between the depths determined by the first and second potential barriers shown in Figure 2 is detected. The calculation was carried out as follows.

The photodiode responsivity *R*, in amperes per watt, to a given wavelength *λ,* is calculated from the external quantum efficiency *QE* by the relationship shown in the following equation.

(3) $$R=QE\times \frac{q\times \lambda }{h\times c},$$

where, *q* is the elementary charge, *h* is the Plank’s constant, and *c* is the speed of light. In turn, *QE* can be calculated from the internal quantum efficiency *QE_int_* and the optical transmittance *T* of the passivation layer(s) deposited over the photodiode, according to the following relation.

(4) $$QE=QE_{int}\times T.$$

Since the passivation layer(s) can be designed independently from the photodiodes, we will focus in *QE_int_* in this work. Brief discussions regarding the transmittance of the passivation layers will be held in Section 3.3. 

Using Equations (1) and (2), it is possible to estimate the photodiodes *QE_int_* by calculating the ratio of light absorption within the first and the second potential barriers. This is performed by subtracting the light intensity in the second potential barrier from the light intensity in the first potential barrier and dividing by the incident light, as follows.

(5) $$QE_{int}=\;e^{-\left(\alpha \;\times \;d1\right)}-\;e^{-\left(\alpha \;\times \;d2\right)},$$

where, *d*_1_ and *d*_2_ are the depth for the first and the second potential barriers of the photodiode, respectively, and *α* is the absorption coefficient of light in silicon for each wavelength. This simplified model for calculation neglects the diffusion of carriers.

**Figure 4:**
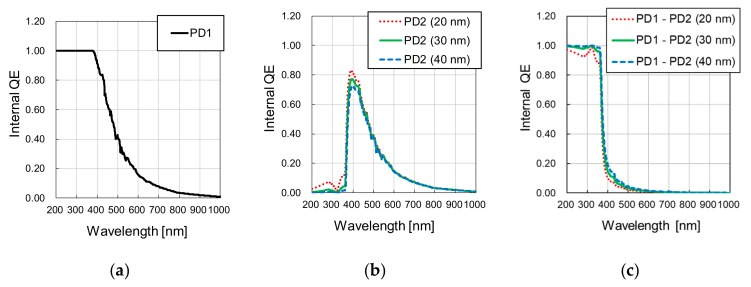
Calculated internal quantum efficiency (QE) for (**a**) PD1, by assuming a first potential barrier at 0 nm depth and a second potential barrier at 460 nm depth, (**b**) PD2, for the conditions of first potential depth of 20 nm, 30 nm and 40 nm, with the second potential depth at 460 nm, and (**c**) PD1 – PD2, for each PD2 condition.

Using Equation (5), the *QE_int_* for PD1 and PD2 were calculated and are shown in Figure 4a,b, for the first potential barrier depth at 0 nm in PD1 and 20 nm, 30 nm and 40 nm in PD2, with the second potential barrier depth at 460 nm in both photodiodes. Figure 4c shows the *QE_int_* of the differential spectral response (PD1 – PD2) calculated by subtracting the *QE_int_* of PD2 from PD1. For those parameters, a high *QE_int_* of almost 100% for wavelengths up to 360 nm is expected, with a sharp *QE_int_* reduction to 50% at 370 nm, 10% at 400 nm and less than 5% at 500 nm. 

In this research, for validation of the proposed principle of the differential spectral response UV sensor, we aimed at a 30 nm depth for the first potential barrier in PD2 during its design. For the second potential barrier in PD1 and PD2, we aimed at 460 nm for visible and NIR responsivity reduction. For applications that require UV-C (190 nm to 280 nm) or UV-B (280 nm to 315 nm) light sensing, such as solar UV-B measurement and flame detection, we propose using the developed differential spectral response sensor with an interference interlayer composed of several stacks of SiO_2_ and SiN thin films, to reshape the sensor’s spectral sensitivity characteristics by using light interference to reduce responsivity in the 315 nm to 500 nm waveband. 

### 2.3. Photodiode Fabrication Process and Device Simulations

**Figure 5:**
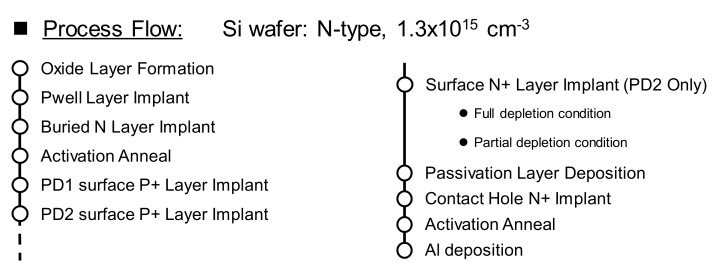
Process flow used in the simulations and manufacturing of the photodiodes.

**Figure 6:**
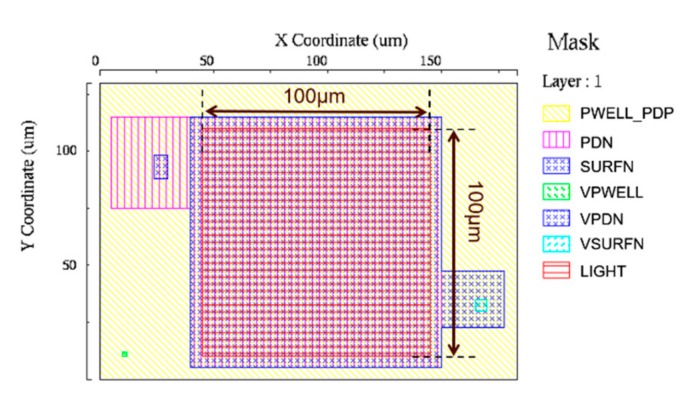
Mask configuration used for PD2 simulation. The central 100 μm × 100 μm area shows the light illumination region used in the spectral sensitivity simulation.

The process flow considered for the proposed photodiodes is shown in Figure 5. It is highly suitable for monolithic integration of PD1 and PD2, and it requires only two added photomasks when compared with conventional photodiodes: one photomask to differentiate the PD1 and PD2 surface P^+^ layers, and another for the surface N^+^ layer ion implant of PD2. This process flow, as well as the layout diagram shown in Figure 6, were used in the 2D process simulator Athena from Silvaco Inc. and the 3D device simulator SPECTRA from Link Research Corporation, to simulate the dopant profiles, surface layer depletion conditions, internal potentials and spectral sensitivities of PD1 and PD2. 

Figure 6 shows the layout used for PD2. PD1 was simulated with a similar layout, but without the surface N^+^ layer mask (SURFN) and its electrode (VSURFN). The explanation of each mask name shown in Figure 6 is summarized in Table 1. Whether or not the surface N^+^ layer of PD2 is depleted was analyzed by simulating the concentrations of donors, acceptors, electrons and holes in the target layers.

In all simulations, a reserve bias of 0.3 V was applied to the electrode for the buried N layer (VPDN), and other electrodes were set to 0.0 V. Also, in the spectral sensitivity simulation, a light irradiation of power 1 × 10^−5^ W/m^2^ was set over the photodiode buried N layer (PDN) mask by using the LIGHT mask, in an area of 100 μm × 100 μm. Then, the steady state photocurrent of the VPDN electrode was simulated for wavelengths in the range of 200 nm to 800 nm, in 10 nm steps. Finally, *QE_int_* was calculated from the obtained electrical current values.

Table 2 summarizes the conditions of three simulations that were carried out, named as sim1, sim2 and sim3, respectively for PD1, PD2 designed to have a fully depleted surface N^+^ layer and PD2 designed to achieve a partially depleted surface N^+^ layer by increasing its dopant concentration. The surface N^+^ layer concentrations used in each simulation are also summarized in Table 2.

The motivation for using two different conditions for the PD2 surface N^+^ layer was to test the effects of full and partial depletion in UV responsivity, due to the difference in the length of electron transit to be drained out. In the full depletion type, photo-electrons must transit to the N^+^ region to be drained out, while in the partial depletion type, relaxation occurs due to the electrons in the neutral region. Therefore, it is expected that PD2 of sim3 achieves lower UV responsivity than PD2 of sim2. If this estimation holds, PD2 of sim3 leads to a better UV responsivity after the differential spectral response extraction. 

**Figure 7:**
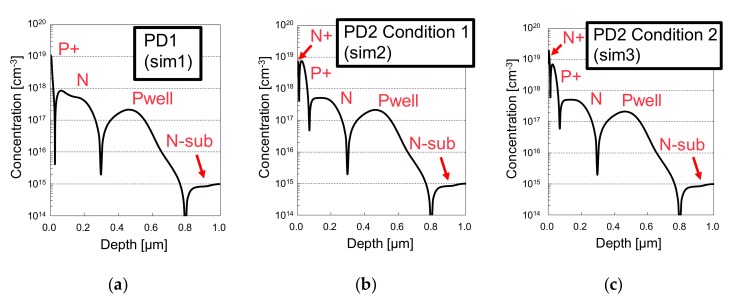
Concentration profiles obtained from the process simulation, where (**a**) shows the PD1 profile, (**b**) shows PD2 profile for full depletion and (**c**) for partial depletion in the surface N^+^ layer.

**Figure 8:**
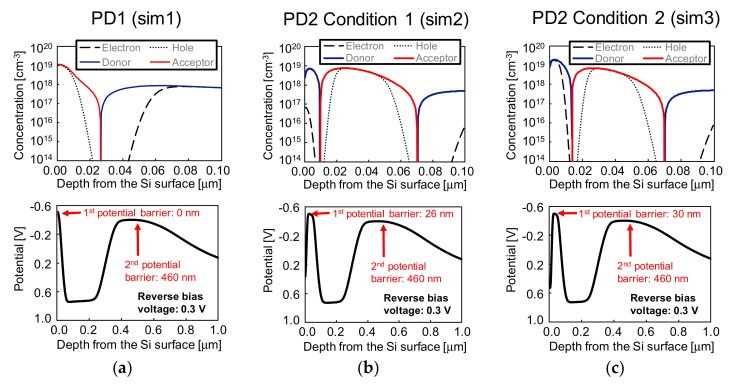
Simulation results for the acceptor, donor, electron and hole concentrations (**top**) and potential diagram (**bottom**) for (**a**) PD1, (**b**) PD2 in the case of full depletion and (**c**) PD2 in the case of partial depletion of the surface N^+^ layer.

The simulation results of the doping profiles for sim1, sim2 and sim3 are shown in Figure 7a–c, respectively. The simulation for acceptor, donor, electron and hole concentrations from the surface to a depth of 0.10 μm, as well as the internal potentials from the surface to a depth of 1.0 μm are shown in Figure 8a–c, for sim1, sim2 and sim3, respectively. By comparing the concentrations of donors and electrons, we can conclude that the PD2 surface N^+^ is fully depleted in sim2 and partially depleted in sim3. It is also clear that the surface P^+^ layer is not fully depleted neither in PD1 nor in both PD2 conditions, by comparing acceptor and hole concentrations. From the potential diagrams, the simulated depth for the first and second potential barriers are 0 nm and 460 nm in PD1, 26 nm and 460 nm in sim2 of PD2, and 30 nm and 460 nm in of sim3 of PD2. The *QE_int_* simulation results of PD1, PD2, and PD1 – PD2 will be presented in the Section 3.1, in comparison with the measurement results. At a simulation level, the difference was within a negligible level between *QE_int_* of PD2 in sim2 and sim3. 

### 2.4. Device Fabrication

**Figure 9:**
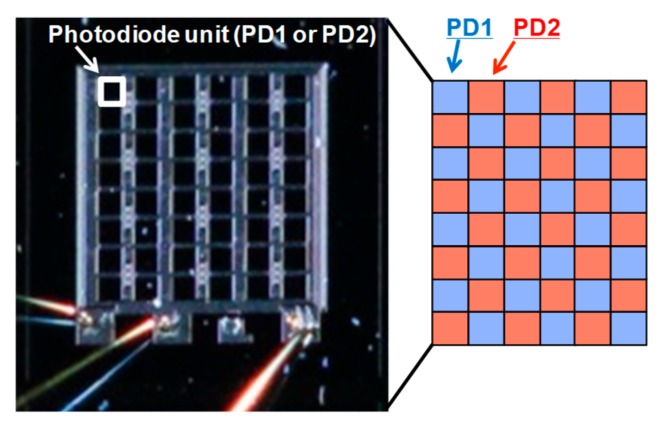
Chip micrograph, showing the employed checker pattern of 8 × 6 photodiode units.

Both types of photodiodes, PD1 and PD2, were monolithically manufactured side by side in a checker pattern, as shown in Figure 9. The checker pattern contains 8 × 6 photodiodes, half of each type. This approach was used in order to spatially average the incident light in each type of photodiode, avoiding errors in the UV detection when non-uniform or oblique incident light is irradiated. It also reduces the effects of process variances, such as differences in the passivation layer thickness on each small PD unit. The fabricated chip size is 1.2 mm × 1.2 mm. The size of each photodiode PD1 and PD2 is 0.12 mm × 0.12 mm. The manufactured samples are summarized in Table 3. Ion implantation process conditions of PD1 is the same in both samples, but the passivation SiO_2_ layer thickness and the PD2 conditions differ.

## 3. Measurement Results and Discussion

This section shows the measurement results of the manufactured photodiodes’ characteristics, such as spectral response and robustness to UV light exposure, and a comparison with the simulation results. The spectral response was measured by a system consisting of the EQ-99 light source, from Energetiq Technology Inc., (Wilmington, MA, USA) and the monochromator SPG-120UV, manufactured by Shimadzu Corporation (Kyoto, Japan). Before each spectral response measurement, the system was calibrated by using a commercially available S1336-18BQ photodiode, manufactured by Hamamatsu Photonics (Hamamatsu City, Japan), to measure the incident radiant power of the system composed by the light source, the optical fiber and the monochromator. Photocurrent was measured using the semiconductor device parameter analyzer B1500A, manufactured by Aligent Technologies, Inc. (Santa Clara, CA, USA). A reverse bias of 0.3 V was applied to the buried N layer for measurement. The robustness to UV light was measured by exposing the developed sensor to the super high-pressure mercury discharge UV lamp USH-250SC, manufactured by Ushio (Tokyo, Japan). 

### 3.1. Spectral Response Measurement and Comparison with Simulation Results

**Figure 10:**
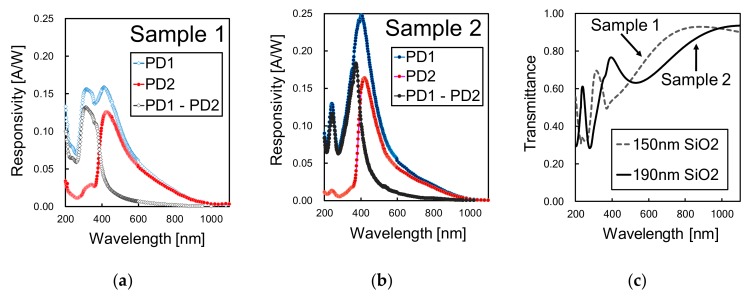
Measurement results of the spectral response for (**a**) Sample 1 and (**b**) Sample 2. (**c**) shows the calculated transmittance characteristics of the employed SiO_2_ passivation layer in each sample.

The measurement results of responsivity, in amperes per watt, for PD1 and PD2 of both samples 1 and 2 are shown in Figure 10a,b, respectively. From the results, it is confirmed that the responsivity selectivity to UV light is improved by extracting the differential spectral response (PD1 – PD2). Figure 10c shows the transmittance of the SiO_2_ passivation layer deposited in each sample, calculated from the SiO_2_ optical constants and the sample’s layer thickness shown in Table 3. The SiO_2_ layer thickness was measured during each sample fabrication with an ellipsometer. As the results show, the passivation layer thickness was not optimized for UV detection, with a rather low transmittance to UV light due to light interference. 

**Figure 11:**
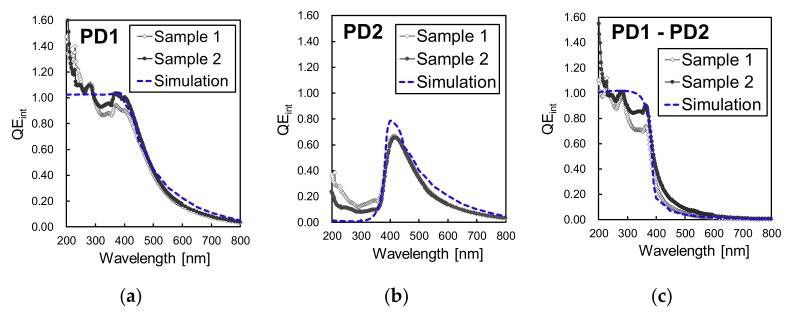
Comparison of the internal quantum efficiency (*QE_int_*) between the measurement results of samples 1 and 2 and the simulation results, for (**a**) PD1, (**b**) PD2 and (**c**) differential spectral response PD1 – PD2.

Since the passivation layer and the photodiodes can be designed and optimized independently, the measured responsivity was converted to *QE_int_* for analysis. For the conversion, we first calculated the *QE* by using the measured responsivity shown in Figure 10a,b with the relationship shown in Equation (3). Then, *QE_int_* was calculated by applying Equation (4) and the SiO_2_ transmittances of Figure 10c. The results for the extracted *QE_int_* are shown in Figure 11a–c for PD1, PD2 and PD1 – PD2, respectively, in comparison with the simulation results.

For PD1, a *QE_int_* similar to the simulation results was obtained, with a difference only for short wavelengths, in the 200 nm to 300 nm waveband, where *QE_int_* of over 1.0 was measured from the manufactured devices. A *QE_int_* higher than 1.0 for short wavelengths is possible because a single high-energetic photon may generate multiple electrons, leading to a quantum yield above 1.0. Discussions about UV light quantum yield can be found elsewhere [25,27,28,29,30,31]. In our calculations, the *QE_int_* was not normalized by the quantum yield of UV light, leading to a *QE_int_* above 1.0 in the measurement results. The simulation results show a maximum *QE_int_* of 1.0 because, in the simulations, the quantum yield effect of high-energetic photons was not considered. Regarding PD2, a residual responsivity to UV light in the 200 nm to 300 nm waveband was detected, with a *QE_int_* of 10% to 40% in sample 1 and of 7% to 23% in sample 2 in this waveband. From the PD2 simulations, less than 2% of residual *QE_int_* for the UV waveband was expected. This difference between the measurement and simulation results may be because of differences in the electron’s lifetime in the surface N^+^ layer of the manufactured device and the simulation settings. Also, from the results, the PD2 sample with partial surface N^+^ depletion (sample 2) showed lower UV responsivity than the sample with a fully depleted surface N^+^ layer (sample 1). Therefore, the relaxation effects of a partially depleted surface N^+^ layer is effective in drifting the excess of electrons generated by UV light. 

### 3.2. UV Light Irradiation Stress Test

For the UV light irradiation stress test, responsivity to the wavelengths of 230 nm, 400 nm and 500 nm was measured before and after continuous UV light irradiation of 10 min, 100 min and 1000 min. The total amount of light exposure after 1000 min was of 1.2 × 10^2^ J/cm^2^, 2.6 × 10^2^ J/cm^2^, 5.2 × 10^2^ J/cm^2^ and 1.1 × 10^3^ J/cm^2^ for the wavelengths of 254 nm, 303 nm, 313 nm and 365 nm, respectively [23].

**Figure 12:**
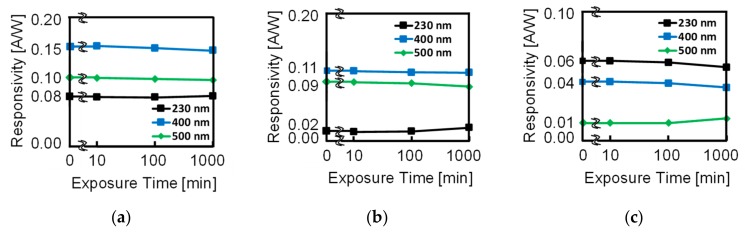
Measurement results of the robustness to UV light for (**a**) PD1, (**b**) PD2 and (**c**) PD1 – PD2.

The results for PD1 and PD2 responsivity to the measured wavelengths after each exposure time are shown in Figure 12a,b, respectively. Figure 12c shows the results for the responsivity in the differential spectral response PD1 – PD2. No significant responsivity degradation due to UV light exposure was detected. 

### 3.3. On-Chip Interference Interlayers as Band-Rejection Filters

**Figure 13:**
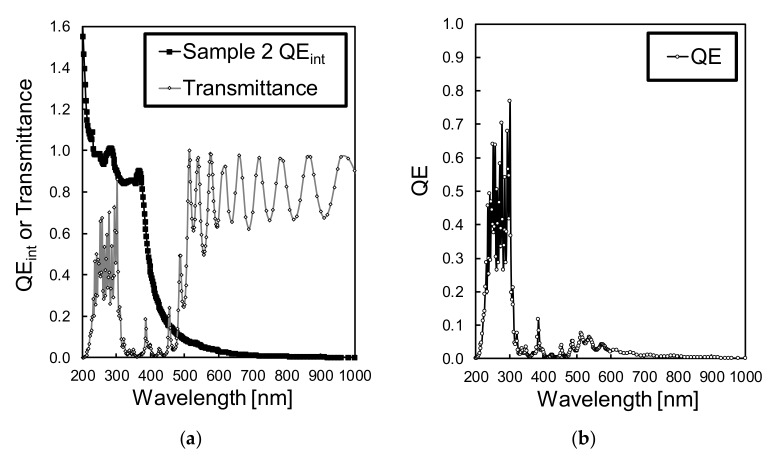
Results for (**a**) transmittance of the interference interlayers, shown with the sample 2 differential *QE_int_*, (**b**) calculated external *QE* expected by multiplying the *QE_int_* in the differential spectral response of sample 2 with the transmittance.

In our previous works, we have proposed, designed and manufactured an interference interlayer consisting of several SiO_2_ and SiN layers with band-rejection properties, capable of reducing light transmittance in the 315 nm to 500 nm waveband range [32]. The interference interlayers are highly suitable to be used with our developed differential spectral response sensor, since the same layer composition can be deposited simultaneously in photodiodes PD1 and PD2. The layer is useful for reshaping the differential spectral response by reducing the residual responsivity to visible light. Figure 13a shows the transmittance of the interference layer, and Figure 13b shows the expected *QE* with the interference interlayer deposition, calculated by the product of the *QE_int_* in the differential spectral response of PD1 and PD2 and the interlayer transmittance, according to Equation (4).

From Figure 13b, by combining our previously reported interference interlayer with the developed differential spectral response sensor of this work, a high selectivity to UV-C and UV-B wavebands can to be obtained, with QEs of 0.40, 0.60, 0.01 and 0.02 for wavelengths of 250 nm, 300 nm, 330 nm and 500 nm, respectively. This approach can be used for applications that require UV-C and UV-B detection under background light conditions, such as flame detection or UV-B measurement in sunlight. Since other transmittance characteristics can be easily obtained by changing the interlayer structure, this method is expected to be adaptable to several UV sensing applications by optimizing both the interlayer and the photodiode structures.

## 4. Conclusions

In this work, we developed a silicon UV radiation sensor with over 90% internal QE to UV light, high selectivity to the UV waveband and high robustness of responsivity towards UV light exposure. The developed sensor utilizes the differential spectral response between photodiodes with high and low UV responsivity, monolithically integrated in a checker pattern containing several photodiodes for spatially averaging incident light. We have also proposed the use of an interference interlayer designed to cut light transmittance from 315 nm to 500 nm by destructive interference, acting as a band-rejection filter. By combining the differential spectral response sensor with the interference interlayer, we estimated that QEs of 0.40, 0.60, 0.01 and 0.02 for wavelengths of 250 nm, 300 nm, 330 nm and 500 nm, respectively, are expected to be obtained. The developed sensor is suitable for UV-C and UV-B light detection, thus applicable for flame detection, as well as for solar UV measurement. Also, the developed sensor responsivity can be reshaped to several UV sensing applications by designing appropriate interference interlayers. 

## Figures and Tables

**Table 1:** Mask setup used in the SPECTRA device simulation as seen in Figure 6.

Mask Name	Function
PWELL_PDP	Pwell and surface P+ layers
PDN	Buried N layer
SURFN	Surface N+ layer
VPWELL	Electrode for PWELL_PDP
VPDN	Electrode for PDN
VSURFN	Electrode for SURFN
LIGHT	Light irradiation area

**Table 2:** Simulation conditions tested.

Simulation Condition	PD Type	Simulation Conditions
sim1	PD1	No surface N^+^ implant
sim2	PD2	Full surface N^+^ depletion, 1.7 × 10^13^ cm^−2^
sim3	PD2	Partial surface N^+^ depletion, 3.4 × 10^13^ cm^−2^

**Table 3:** Samples manufactured for evaluation and comparison with simulation results.

Sample	Type	PD1 and PD2 Integration	PD2 Condition	SiO_2_ Thickness
1	8 × 6 checker pattern	Monolithically	Full depletion	150 nm
2		Monolithically	Partial depletion	190 nm
